# Iterative and discrete reconstruction in the evaluation of the rabbit model of osteoarthritis

**DOI:** 10.1038/s41598-018-30334-8

**Published:** 2018-08-13

**Authors:** Juuso H. Ketola, Sakari S. Karhula, Mikko A. J. Finnilä, Rami K. Korhonen, Walter Herzog, Samuli Siltanen, Miika T. Nieminen, Simo Saarakkala

**Affiliations:** 1 Research Unit of Medical Imaging, Physics and Technology, University of Oulu, Oulu, Finland; 2 Infotech Doctoral Program, University of Oulu, Oulu, Finland; 3 Medical Research Center, University of Oulu and Oulu University Hospital, Oulu, Finland; 4 Department of Applied Physics, University of Eastern Finland, Kuopio, Finland; 5 Human Performance Laboratory, Faculty of Kinesiology, University of Calgary, Calgary, AB Canada; 6 McCaig Institute for Bone and Joint Health, University of Calgary, Calgary, AB Canada; 7 Department of Mathematics and Statistics, University of Helsinki, Helsinki, Finland; 8 Department of Diagnostic Radiology, Oulu University Hospital, Oulu, Finland

## Abstract

Micro-computed tomography (µCT) is a standard method for bone morphometric evaluation. However, the scan time can be long and the radiation dose during the scan may have adverse effects on test subjects, therefore both of them should be minimized. This could be achieved by applying iterative reconstruction (IR) on sparse projection data, as IR is capable of producing reconstructions of sufficient image quality with less projection data than the traditional algorithm requires. In this work, the performance of three IR algorithms was assessed for quantitative bone imaging from low-resolution data in the evaluation of the rabbit model of osteoarthritis. Subchondral bone images were reconstructed with a conjugate gradient least squares algorithm, a total variation regularization scheme, and a discrete algebraic reconstruction technique to obtain quantitative bone morphometry, and the results obtained in this manner were compared with those obtained from the reference reconstruction. Our approaches were sufficient to identify changes in bone structure in early osteoarthritis, and these changes were preserved even when minimal data were provided for the reconstruction. Thus, our results suggest that IR algorithms give reliable performance with sparse projection data, thereby recommending them for use in µCT studies where time and radiation exposure are preferably minimized.

## Introduction

Micro-computed tomography (µCT) has long been considered as the ‘gold standard’ method for structural bone analysis due to its ability of retrieving high-resolution volumetric data in a non-invasive manner, and providing optimal contrast between bone and soft tissue^1^. Other applications of µCT include small animal imaging for phenotyping of disease models, evaluating pre-clinical study outcomes, and developing of drug and treatment interventions^2,3^. The imaging times in µCT vary from a few minutes for *in vivo* scans to several hours for high-resolution (<2 µm) *in vitro* scans. Long scan times are associated with high radiation exposure and movement induced imaging artifacts^4,5^. In extreme cases, the radiation dose is so high that it affects tissue metabolism and produces unwanted tissue changes. Such changes are of special concern in longitudal *in vitro* studies^6^, for example in digital volume correlations used for *in situ* mechanical testing^7^. Furthermore, harmful radiation effects, such as burns, radiation sickness, or cancer, are of scientific and ethical concern^2,3,6^.

In µCT, a cone-beam X-ray geometry and a radiation detector are used to gather projected images from different angles around the test object^2,3,8^. These images are reconstructed mathematically to obtain the 3-dimensional structure within the object. Traditionally, reconstructions are made using methods that are based on filtered back-projection (FBP), such as the three-dimensional cone-beam reconstruction algorithm developed by Feldkamp, Davis and Kress in 1984 (FDK)^9^. FBP- or FDK-based methods are robust and computationally efficient but are limited in that they require vast amounts of X-ray projection data, otherwise image reconstructions are plagued by imaging artefacts, such as streaking, stretching, blurring, partial volume effects, low resolution, or noise^4,10^.

CT reconstruction is an inverse problem. Projection data measured with a known X-ray setup (forward problem) are used to retrieve the attenuation information from within the imaged object (inverse problem). With the help of inversion mathematics, images of sufficiently good quality (minimal artefacts and noise) can be computed iteratively from a limited amount of attenuation data^10–12^, reducing the imaging time and radiation dose^12,13^. Simple iterative reconstruction (IR) algorithms, such as the simultaneous algebraic reconstruction technique (SART)^14^, or simultaneous iterative reconstruction technique (SIRT)^15^, use an algebraic solver to minimize the Euclidean norm, L_2_, of the residual of the reconstruction. By using iterative techniques, the radiation dose can be reduced up to 76% while still obtaining image quality comparable to FBP-based methods^11^. Accordingly, a better image quality can be achieved by using IR instead of FBP with the same amount of radiation dosage^13^. The inverse problems associated with sparse X-ray tomography data reconstructions often do not have stable and unique solutions and require *a priori* knowledge about the object to converge to a reliable solution^16,17^. This so-called regularization consists of iterative algorithms aimed at solving an optimization problem (*e.g*. the minimization of the L_2_ norm) with a penalty term containing prior information. Some well-known examples are the Tikhonov regularization and the total variation (TV) regularization, which take advantage of information about the smoothness and edges within the target object^17^. In the context of µCT imaging, IR or regularizing methods could enable the acquisition of a large number of high-resolution scans, thereby increasing reliability of many biologically relevant µCT studies while simultaneously reducing movement artefacts and harmful radiation exposure. However, due to their iterative nature, these algorithms are associated with high computational demands. However, the computational power of commercially available workstations has increased to a level where IR is a viable option, and the major clinical CT vendors have already implemented IR algorithms into their imaging systems^10,13^. In clinical applications, IR techniques are usually applied with a certain amount of FBP to reduce blotchy image appearance obtained with full IR^11,13^. Further reasons why IR is not more widely used in clinical CT include the limited accessibility to corrected raw data, the problems with intellectual property rights, and the communication barrier between mathematicians and engineers^18^. With high-performance GPUs, IR can be performed even for high-resolution µCT datasets that are considerably larger than those obtained in clinical CT.

Since µCT achieves high spatial resolution and contrast between bone and soft tissues, it has become an important tool for various musculoskeletal diseases, such as osteoarthritis (OA). OA is a progressive joint disease causing pain and stiffness in synovial joints, and may cause disability in middle aged and old people. Risk factors for OA include age, excessive weight, joint instability, mechanical impacts, large torsional loads, and injury^19^. Because OA is a progressive disease with little or no symptoms in its early stage, only severe and symptomatic cases are usually studied in human patients. Different animal models have been studied alongside the human disease to obtain a better understanding of OA^20–23^. OA is associated with degradation of the extracellular matrix and changed chondrocyte function, causing degeneration and disturbed repair of articular cartilage^19,24,25^. OA-related damage is not only present in the articular cartilage, but also the subchondral bone which also undergoes structural changes with increasing OA severity^26^. Osteoarthritic trabecular bone often has coarser and discontinuous microstructure, resulting in decreased stiffness, which can be quantified by changes in structural parameters such as trabecular bone volume fraction, and the thickness and separation of trabeculae^27–29^.

The standard method of obtaining quantitative information on bone structure is via quantitative bone morphometry, in which bone data acquired with µCT is analyzed mathematically^30,31^. First, the data are segmented into bone and other tissues, after which morphometric parameter analyses are performed to obtain information about the size and shape of specific bone compartments. The minimal set of variables to be reported in such analyses are bone volume fraction (BV/TV), trabecular number (Tb.N), trabecular thickness (Tb.Th) and trabecular separation (Tb.S)^32^. Along with these parameters, structural model index (SMI) has also been calculated to quantify as a degree of anisotropy in trabecular structure. However, it is known that SMI has limitations, such as not accounting for concave bone surfaces^33^, and recently the ellipsoid factor (EF) has been introduced in its place with convincing results^34^. In the light of these findings, EF is used instead of SMI in this study to assess the geometric composition of our bone samples. Furthermore, the thickness of the subchondral bone plate (Pl.Th) is also quantified. This set of parameters provides a quantitative overview of the structure of the bone and how it changes with the progression of OA.

The aim of this study was to test the performance of different image reconstruction algorithms in a µCT setting. The algorithms are tested using quantitative parameter analysis related to the biomedical imaging studies of OA, and the results are compared against those given by FDK reconstruction.

## Materials and Methods

### Sample acquisition

Six female New Zealand white rabbits (Oryctolagus cuniculus, age 14 months at the end of the experiments, weight 4.5 ± 0.3 kg) underwent unilateral anterior cruciate ligament transection (ACLT) surgery to a randomized knee to induce the onset of OA. A control group (n = 4, age 14 months at the end of the experiments, weight 4.8 ± 0.3 kg) was left unoperated. After 8 weeks, the animals were euthanized and the femoral condyles (both medial and lateral) were harvested from the operated knees of the ACLT group and both knees of the control group. All procedures were approved by the Committee on Animal Ethics at the University of Calgary and were carried out according to the guidelines of the Canadian Council on Animal Care (certificate of animal use protocol approval #AC11-0035). Femoral condyles were wrapped in moisturized tissue paper and placed in a plastic container to prevent drying and movement, respectively. From these samples (N = 12 and N = 16 for ACLT and control groups, respectively), subchondral bone properties were analyzed quantitatively using different image reconstruction algorithms. The medial and lateral condyles were pooled for statistical power because of the small number of samples.

### Micro-computed tomography

Projection data were acquired using a SkyScan 1272 high-resolution cone-beam µCT scanner (Bruker micro-CT, Kontich, Belgium) with 50 kV source voltage, 200 µA source current, focal spot size of <5 µm at 4 W power, and additional 0.5 mm aluminum filtering. Each projection image was taken with 2016 ms exposure time, 4 × 4 binning, and 2 frames (rays) per projection averaging. Datasets of 260 projection images were measured from 181.3° angle of view with 0.7° angular step size and isotropic 9 µm camera pixel size. The image size of projections was 1008 × 672.

### Image reconstruction

The tomographic datasets were reconstructed into a 500 × 500 × 600 volume of interest (VOI) with isotropic 25 µm voxel side length in several ways. First, an FDK reconstruction (Hamming filter, α = 0.54) of the full (260 projections) dataset was obtained. Then, the projection data was reduced to half, one-fourth and approximately one-sixth of the original amount of the projection images (130, 65 and 44 projections, respectively). All projection data were pre-processed in the NRecon software (Bruker micro-CT, Kontich, Belgium) to correct for beam hardening, post-alignment and ring artifacts for improved image quality. The reduced datasets were then reconstructed with three different iterative reconstruction methods; the least squares implementation of the conjugate gradient method (CGLS)^35^, the total variation regularization method (TV)^36^, and the discrete algebraic reconstruction method (DART)^37^. The CGLS algorithm was chosen for its computational efficiency and applicability in solving large-scale linear systems^38^. TV regularization was chosen because of its edge-preserving properties that can be useful in X-ray tomography^36,39^, and the Barzilai-Borwein minimization^40^ was used for large scale TV regularization. The regularization parameter was chosen with the L-curve method^41^, which was supported by visual evaluation of image quality. The DART algorithm was chosen because it incorporates *a priori* information about the grayscale values of the data in the reconstruction. As bone samples have highly distinguishable values for different materials, such knowledge can be powerful when working with severely limited amount of projection data^37,42^. The *a priori* grayscale and thresholds values for bone, soft tissue and background classification were chosen manually from the histograms of the corresponding CGLS reconstructions. In each DART iteration, a pre-set amount of iterations of a chosen algebraic reconstruction method (ARM) is run, after which the algorithm performs segmentation and fixation steps to restrict the boundaries of the next update^37^. We chose the ARM to be CGLS with 10 iterations, and 25 CGLS iterations were used to obtain the initial reconstruction. All reconstruction methods were run with 25 base iterations.

All reconstructions were calculated in the MATLAB 2016b programming environment (MathWorks, Natick, MA, USA) with the help of the ASTRA toolbox (iMinds-Vision Lab, University of Antwerp, Belgium)^43–45^, and the Spot toolbox^46^. ASTRA provides a programming and simulation environment for various CT geometries, as well as some popular image reconstruction functions and algorithms. Furthermore, the Spot toolbox and the opTomo operator of ASTRA wrap linear operators (such as forward- and back-projection in computed tomography) to MATLAB objects, resulting in fast and efficient computation with GPU memory^47^. Image reconstruction in the aforementioned frameworks was implemented as follows: (1) generation of projection and volume geometries, (2) computation of the system matrix as per the previously generated geometry objects, (3) running the chosen optimization algorithm on the given projection data, and (4) retrieval of reconstructed image data for further analysis.

Algorithm runtimes were measured in MATLAB as the time it takes for each reconstruction function to perform its computation, omitting the time needed for loading data and preprocessing. As a measure of absolute image quality, contrast-to-noise ratio (CNR) was calculated from the middle slice of the datasets by subtracting the mean value of a background region of interest (ROI) from a homogeneous bone ROI and dividing the result with the standard deviation of the background ROI.

### Segmentation and image processing

The reconstructed µCT data were analyzed in the CT Analyzer (CTAn, v.1.16) software (Bruker micro-CT, Kontich, Belgium). From the data, 2 × 2 × 4 mm^3^ (80 × 80 × 160 voxels) VOIs were chosen from the weight-bearing regions of the femoral condyles, similarly to what has been done previously in the rabbit ACLT model^22^. Trabecular bone and subchondral bone plate regions were manually segmented to obtain masks for parameter analyses. Bone tissue was segmented from other tissues in the µCT data using an automated Otsu thresholding algorithm in 3D^48^. Prior to the thresholding, the images were processed with median (radius = 1) and unsharping (radius = 1, amount = 50%) filters, and after thresholding a despeckling filter (sweep in 3D) was applied to the images to include only the largest object in the volume. Furthermore, for subchondral bone plate thickness analyses, another despeckling filter was run to remove the pores within the plate, and the pores in the edges of the plate were removed with morphological closing.

### Quantitative bone morphometry

After the pre-processing, the structural parameters (Table 1) were calculated from the processed images. All computations were performed with direct 3D methods in CT Analyzer, except for the ellipsoid factor (EF), which was calculated from the binarized image stacks with the BoneJ plugin (v.1.4.2)^49^ for the ImageJ software (v. 1.51n, National Institutes of Health, Bethesda, Maryland, USA)^50^. Furthermore, all of the calculated bone parameter values were statistically tested using Mann-Whitney testing for two independent datasets to see whether there were statistically significant differences between the ACLT and control groups. Mann-Whitney non-parametric testing was chosen because of the small sample sizes, and as it does not require assumption of normally distributed data. In addition, the relative errors (%) of the parameters calculated from IR data to the parameters calculated from the reference FDK reconstruction were calculated and averaged for each algorithm and sparsity level. All statistical analyses were performed using SPSS software (v.24, IBM Analytics, New York, USA).

**Table 1:** Structural bone parameters calculated from µCT data, with abbreviations, base units and definitions.

Parameter		Unit	Definition
**Subchondral bone plate**			
Plate thickness	(Pl.Th)	µm	Average thickness of the subchondral bone plate
**Trabecular bone**			
Bone volume fraction	(BV/TV)	%	Number of pixels classified as bone divided by total amount of pixels
Trabecular thickness	(Tb.Th)	µm	Mean thickness of trabeculae
Trabecular separation	(Tb.S)	µm	Mean thickness of the spaces between trabeculae
Trabecular number	(Tb.N)	mm^−1^	Linear density of trabeculae, *i.e*. amount of trabeculae per unit length
Ellipsoid factor	(EF)	(a.u.)	Measures the anisotropy in the data by determining how prolate (rod-like) or oblate (plate-like) the trabeculae are in the sample. Negative EF values correspond to prolate and positive values to oblate dominancy in the geometry.

## Results

In the reference FDK reconstruction, BV/TV, Tb.Th, Tb.N and EF were lower in the ACLT group compared to the control group, and Pl.Th and Tb.S were higher in the ACLT group compared to the control group (Table 2). Three of these parameters (BV/TV, Tb.Th and EF) were significantly different.

**Table 2:** Descriptive statistics (mean ± standard deviation) of calculated parameters in the ACLT (N = 12) and Control (N = 16) groups (data reconstructed with FDK and all projections), and the statistical differences within them. Statistical difference was tested with non-parametric Mann-Whitney testing (exact), *p*-values listed as one-tailed (two-tailed).

Parameter		ACLT	Control	*p*-values
**Subchondral bone plate**				
Pl.Th	(µm)	531.8 ± 90.7	485.0 ± 99.7	0.268 (0.537)
**Trabecular bone**				
BV/TV	(%)	47.7 ± 4.4^**^	51.9 ± 3.4	0.010 (0.020)
Tb.Th	(µm)	202.9 ± 18.2^*^	214.7 ± 16.1	0.045 (0.090)
Tb.S	(µm)	311.6 ± 45.0	285.6 ± 25.9	0.080 (0.159)
Tb.N	(mm^−1^)	2.36 ± 0.19	2.42 ± 0.12	0.254 (0.507)
EF	(a.u.)	−0.189 ± 0.04^**^	−0.266 ± 0.04	0.001 (0.002)

^*^Values significantly different from the Control group (one-tailed p < 0.05). ^**^Values significantly different from the Control group (two-tailed p < 0.05).

**Figure 1:**
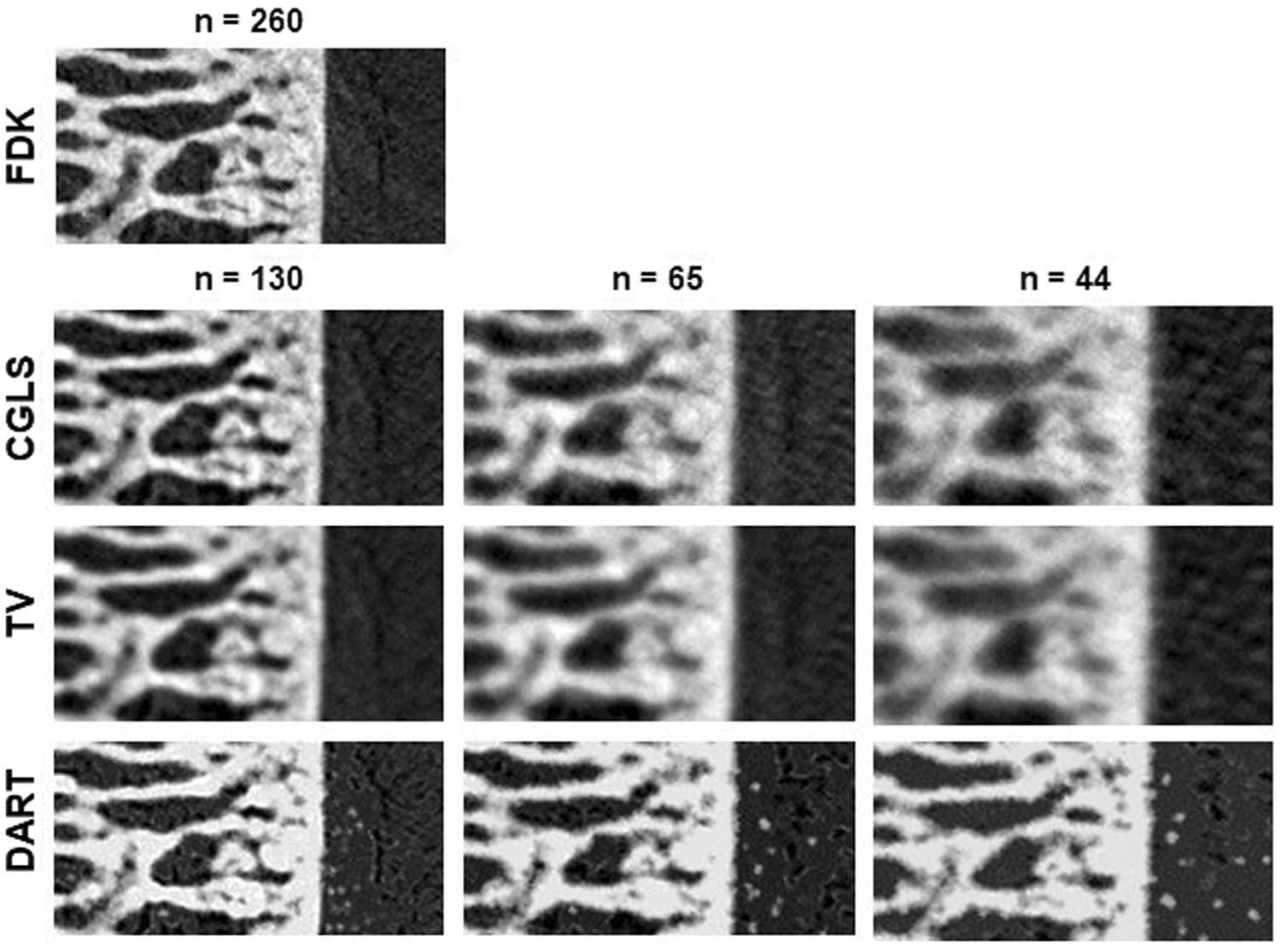
Example images of reconstructed data, shown as one 80 × 160 pixel image from the 80 × 80 × 160 voxel volume of interest. FDK = Feldkamp, David and Kress algorithm, CGLS = conjugate gradient least squares algorithm, TV = total variation regularization and DART = discrete algebraic reconstruction technique. The number of projection images used in reconstruction is denoted by n.

**Figure 2:**
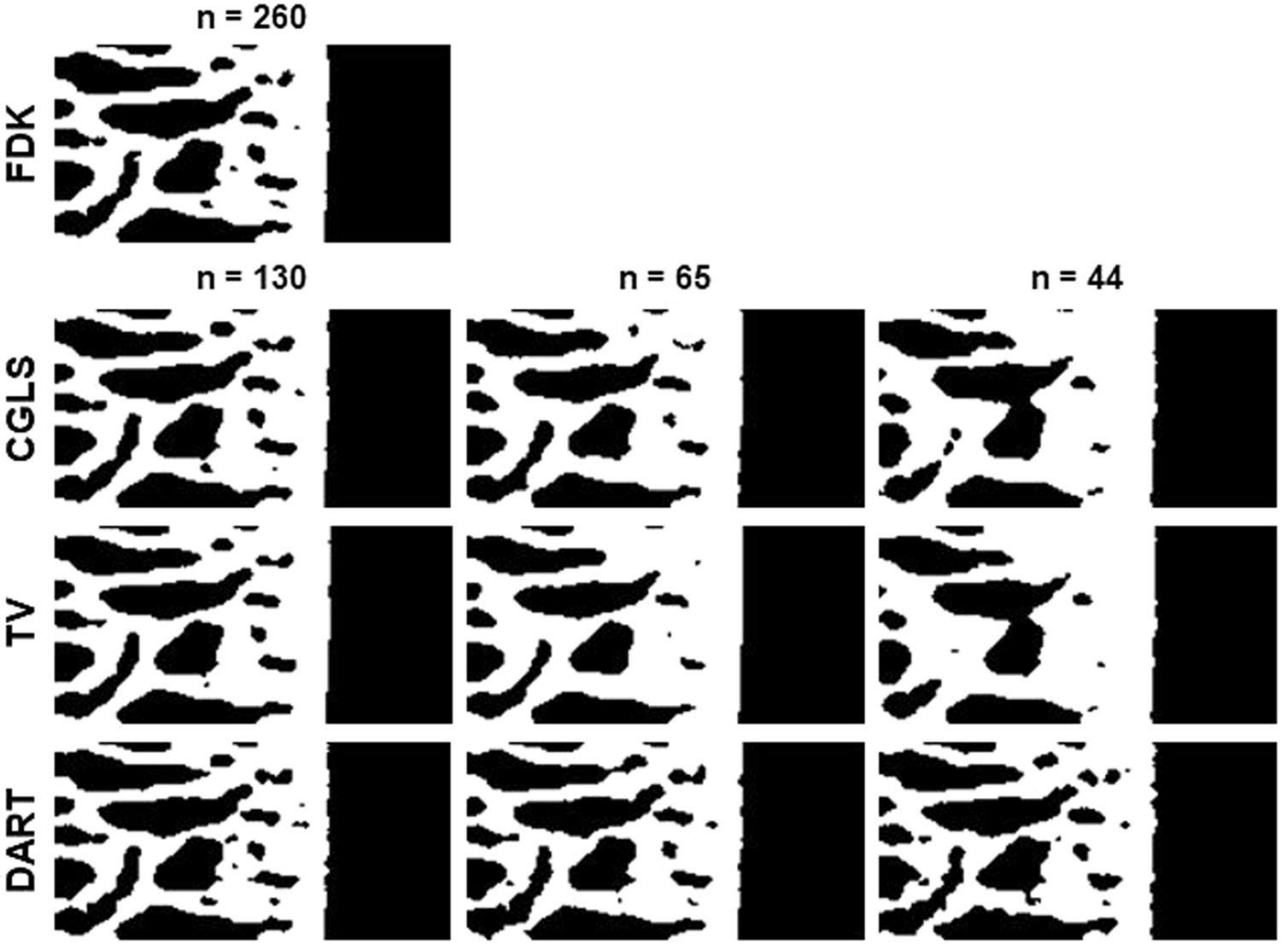
The binarized slice images corresponding to the data in Fig. 1. FDK = Feldkamp, David and Kress algorithm, CGLS = conjugate gradient least squares algorithm, TV = total variation regularization and DART = discrete algebraic reconstruction technique. The number of projection images used in reconstruction is denoted by n.

Results obtained for CGLS, TV and DART are presented in Table 3. Out of the iterative algorithms, DART performed best when compared to the results of the full data FDK reconstruction for all reduced datasets. CGLS and TV performed well with half of the original projections, but further reduction resulted in great differences from the reference bone parameter values. DART was the only iterative algorithm that preserved the statistical significance in BV/TV, Tb.Th and EF across all reduced datasets. Example reconstructions of all algorithms are presented in Fig. 1, and the segmented binary images of those reconstructions (used for parameter analyses) are presented in Fig. 2.

**Table 3:** Descriptive statistics (mean ± standard deviation) of calculated parameters in the ACLT (N = 12) and Control (N = 16) groups, and the statistical differences within them.

**Parameter**		**ACLT**	**Control**	***p*** **-values**	**ACLT**	**Control**	***p*** **-values**	**ACLT**	**Control**	***p*** **-values**
**1/2 of full data**		**CGLS**			**TV**			**DART**		
**Subchondral bone plate**										
Pl.Th	(%)	531.5 ± 92.6	483.3 ± 99.6	0.254 (0.508)	533.2 ± 93.3	486.2 ± 97.9	0.254 (0.508)	527.6 ± 90.9	484.6 ± 99.4	0.316 (0.631)
**Trabecular bone**										
BV/TV	(%)	47.9 ± 4.3**	51.7 ± 3.4	0.019 (0.037)	49.8 ± 4.3^*^	53.2 ± 3.2	0.026 (0.053)	47.6 ± 4.8**	51.9 ± 3.7	0.015 (0.029)
Tb.Th	(µm)	202.8 ± 16.4	213.7 ± 16.4	0.061 (0.121)	220.5 ± 16.8	228.4 ± 17.2	0.140 (0.280)	199.2 ± 18.5^*^	213.1 ± 17.6	0.033 (0.066)
Tb.S	(µm)	308.2 ± 45.8	283.8 ± 25.7	0.111 (0.223)	311.7 ± 45.2	287.9 ± 24.0	0.087 (0.174)	305.8 ± 41.5	281.7 ± 25.9	0.095 (0.189)
Tb.N	(mm^−1^)	2.37 ± 0.18	2.42 ± 0.12	0.265 (0.529)	2.26 ± 0.17	2.34 ± 0.12	0.148 (0.296)	2.40 ± 0.18	2.44 ± 0.11	0.353 (0.706)
EF	(a.u.)	−0.201 ± 0.06**	−0.268 ± 0.05	0.002 (0.004)	−0.203 ± 0.05**	−0.277 ± 0.05	0.002 (0.004)	−0.189 ± 0.05**	−0.263 ± 0.05	0.001 (0.002)
**1/4 of full data**		**CGLS**			**TV**			**DART**		
**Subchondral bone plate**										
Pl.Th	(%)	540.2 ± 93.6	490.2 ± 98.1	0.211 (0.423)	543.3 ± 93.9	495.2 ± 96.5	0.186 (0.371)	524.6 ± 94.8	486.86 ± 97.9	0.332 (0.664)
**Trabecular bone**										
BV/TV	(%)	52.0 ± 4.3**	55.4 ± 3.3	0.024 (0.047)	53.8 ± 4.8	57.1 ± 3.5	0.055 (0.110)	47.4 ± 4.2**	52.6 ± 4.2	0.002 (0.004)
Tb.Th	(µm)	236.7 ± 16.0	243.1 ± 17.4	0.186 (0.371)	255.4 ± 19.9	260.2 ± 18.5	0.316 (0.631)	202.8 ± 18.3**	219.3 ± 17.9	0.021 (0.042)
Tb.S	(µm)	306.4 ± 45.9	281.6 ± 24.6	0.073 (0.146)	314.9 ± 46.0	289.1 ± 25.2	0.103 (0.205)	304.5 ± 46.1	276.4 ± 27.4	0.061 (0.121)
Tb.N	(mm^−1^)	2.20 ± 0.14	2.28 ± 0.11	0.080 (0.159)	2.11 ± 0.14^*^	2.20 ± 0.12	0.045 (0.090)	2.34 ± 0.19	2.40 ± 0.11	0.198 (0.397)
EF	(a.u.)	−0.205 ± 0.04**	−0.280 ± 0.04	0.001 (0.002)	−0.190 ± 0.05**	−0.280 ± 0.04	0.001 (0.002)	−0.187 ± 0.04**	−0.253 ± 0.04	0.001 (0.002)
**1/6 of full data**		**CGLS**			**TV**			**DART**		
**Subchondral bone plate**										
Pl.Th	(%)	548.4 ± 96.3	498.3 ± 93.7	0.130 (0.260)	551.9 ± 96.8	501.4 ± 92.9	0.130 (0.260)	524.2 ± 95.7	488.9 ± 97.1	0.349 (0.698)
**Trabecular bone**										
BV/TV	(%)	54.4 ± 5.8	58.5 ± 5.0	0.055 (0.110)	55.6 ± 6.4	59.9 ± 5.5	0.061 (0.121)	47.5 ± 4.0**	52.6 ± 4.2	0.002 (0.004)
Tb.Th	(µm)	270.3 ± 24.4	276.5 ± 47.1	0.332 (0.664)	285.6 ± 28.2	291.5 ± 27.5	0.437 (0.873)	202.1 ± 15.4**	219.2 ± 18.3	0.010 (0.020)
Tb.S	(µm)	321.0 ± 47.1	294.1 ± 27.9	0.130 (0.260)	328.1 ± 48.4	299.8 ± 29.9	0.120 (0.241)	301.8 ± 43.6	277.9 ± 28.2	0.095 (0.189)
Tb.N	(mm^−1^)	2.02 ± 0.15^*^	2.12 ± 0.13	0.041 (0.082)	1.95 ± 0.15**	2.05 ± 0.12	0.021 (0.042)	2.35 ± 0.18	2.40 ± 0.12	0.268 (0.537)
EF	(a.u.)	−0.179 ± 0.04**	−0.234 ± 0.05	0.001 (0.002)	^−^0.180 ± 0.05**	−0.252 ± 0.04	0.001 (0.002)	−0.182 ± 0.04**	−0.240 ± 0.05	0.002 (0.004)

Used algorithm and fraction of full projection data used to reconstruct different datasets denoted in the headers. Statistical difference was tested with non-parametric Mann-Whitney testing (exact), *p*-values listed as one-tailed (two-tailed). ^*^Values significantly different from the Control group (one-tailed p < 0.05). ^**^Values significantly different from the Control group (two-tailed p < 0.05).

**Figure 3:**
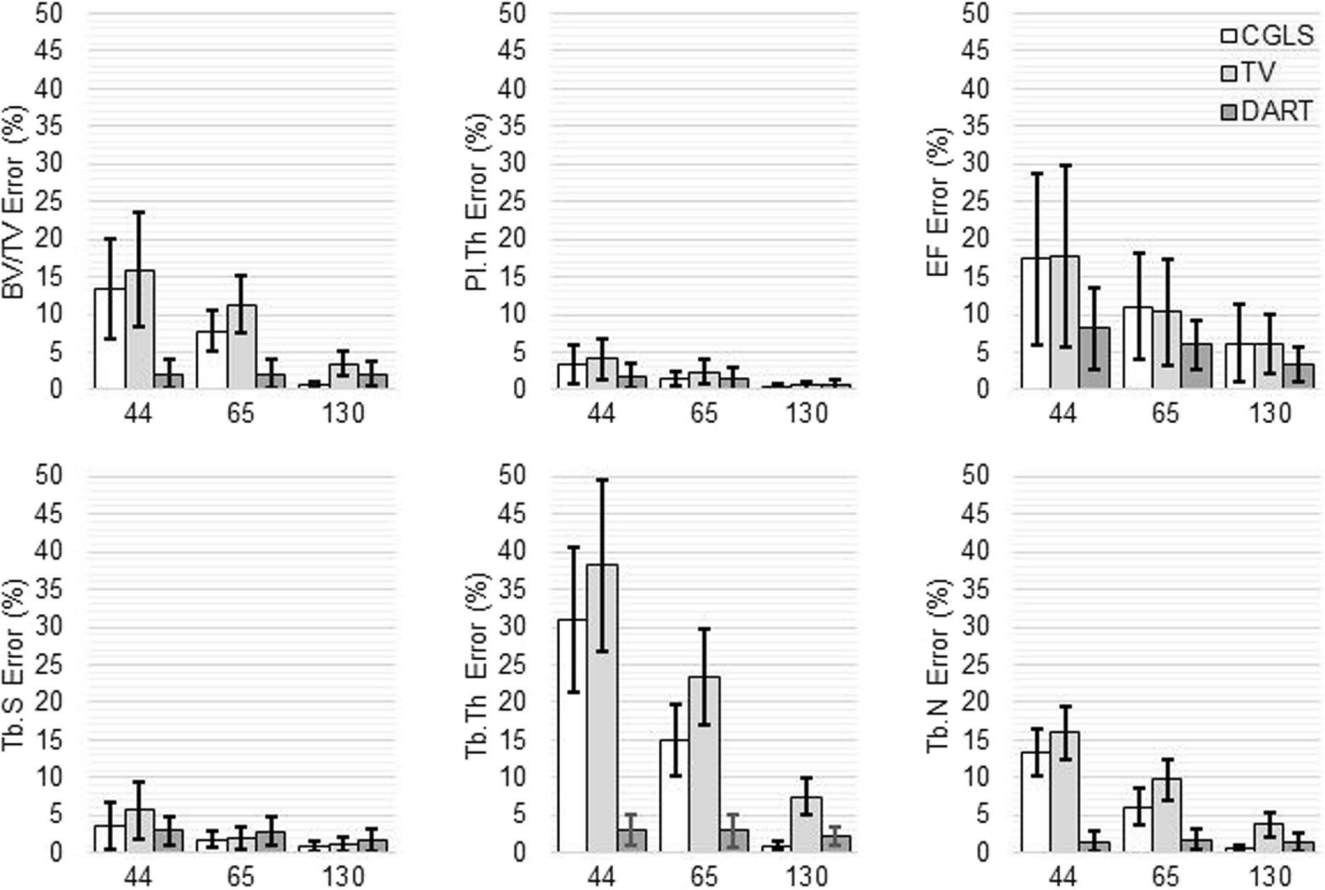
The mean relative error of the used iterative algorithms with regards to reference data in quantitative bone morphometry analysis. The number below each bar trio corresponds to the number of used projection images. The bar height indicates the mean and the error bars indicate the standard deviation of the data. The analyzed morphometric parameters were BV/TV = bone volume fraction, Pl.Th = plate thickness, EF = ellipsoid factor, Tb.S = trabecular separation, Tb.Th = trabecular thickness and Tb.N = trabecular number. FDK = Feldkamp, David and Kress algorithm, CGLS = conjugate gradient least squares algorithm, TV = total variation regularization and DART = discrete algebraic reconstruction technique.

Mean relative errors per algorithm and parameter are presented in Fig. 3. Out of the three algorithms, DART performed best with little errors (2–8%) across all reduced data. Although CGLS and TV gave slightly better results for some parameters with half of the projection data, DART was far superior with reductions to one-quarter or one-sixth of the original data.

**Figure 4:**
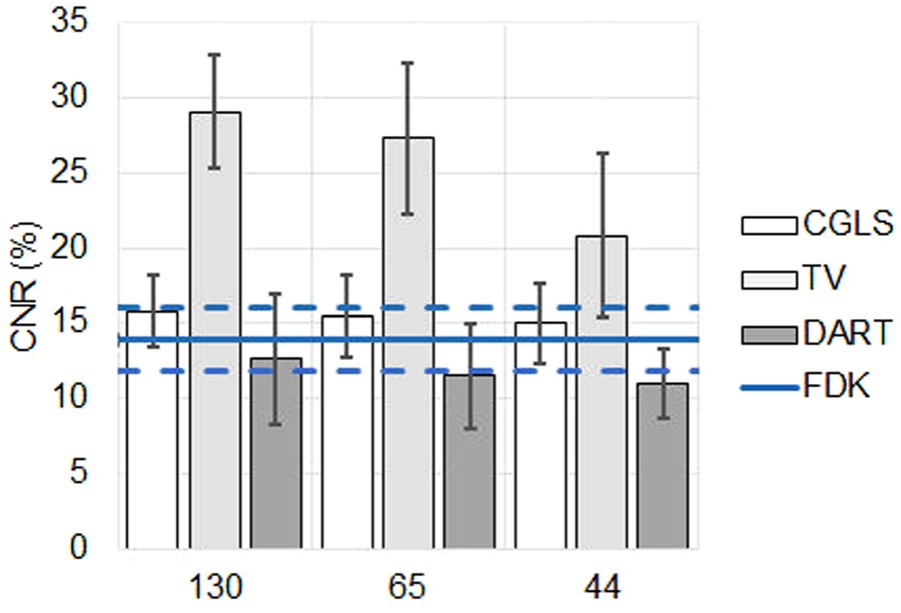
Contrast-to-noise ratio (CNR) as a function of used projection images. For iterative algorithms, the bar height indicates the mean and the error bars indicate the standard deviation in the data. For FDK, the continuous line refers to the mean (reference level) and the dashed lines indicate the standard deviation in the data. FDK = Feldkamp, David and Kress algorithm, CGLS = conjugate gradient least squares algorithm, TV = total variation regularization and DART = discrete algebraic reconstruction technique.

Mean CNR values and their standard deviations are presented in Fig. 4. Across all reduced datasets, TV had the highest value of CNR. For CGLS, CNR was slightly better than the reference (FDK) value, and for DART, it was slightly worse.

Finally, the algorithm runtimes are presented in Table 4. DART and TV had longer runtimes than CGLS. Furthermore, reduction of projection data did not reduce the reconstruction times of TV as efficiently as it did for DART and CGLS.

**Table 4:** Mean values of algorithm runtimes for each algorithm and number of projections (N).

Algorithm	N	Runtime (s)
FDK	260	28.8
CGLS	130	45.2
	65	23.9
	44	15.9
TV	130	1040
	65	966
	44	949
DART	130	1080
	65	638
	44	535

## Discussion

ACLT in rabbits resulted in statistically significant alterations of the subchondral bone structure, indicative of OA, after 8 weeks of surgery. Reduction of bone volume, thinning of trabeculae, and a shifting of trabecular alignment to more oblate configuration were statistically significant. Furthermore, thickening of the subchondral bone plate, increase of trabecular separation, and a reduction in trabecular number were observed, although these changes were not statistically significant. In Florea *et al*.^22^ similar changes in bone volume, trabecular thickness, and trabecular separation were seen in rabbits after 4 weeks of ACLT surgery. However, unlike in our study, the subchondral bone plate was thinned significantly in the medial side and SMI was analyzed instead of EF. Apart from that, our findings on changes to the trabecular bone agree with previous observations in the rabbit model of OA^22,51^.

Out of the three algorithms tested in this study, DART performed best when compared to the reference data, as it gave good results even with severely reduced projection dataset. DART incorporates *a priori* information about the grayscale distribution of the data by strong enforcement of a pre-specified finite set of attenuation values that are the only possible ones in the target of imaging. Actually, very few optimization algorithms for tomographic reconstruction implement this, although other optimization algorithms exist as well in the field of discrete tomography. In our case, the grayscale values in bone datasets can be pre-classified into three values: background, soft tissue (or water), and bone, by just looking at the dataset’s histogram. Therefore, it is possible to estimate the *a priori* values for the aforementioned materials from a test reconstruction, although an automated procedure for estimation exists as well^52^. The easiest of the methods to implement, CGLS, does not use any *a priori* information but only minimizes the L_2_-norm of the residual with a gradient-based optimization algorithm. Therefore, one would not expect it to give good results with highly reduced datasets, even though it showed good performance with only half of the projection data, and is expected to perform really well with a complete projection dataset. The third algorithm, TV regularization, assumes that the *total variation* of the grayscale values in the final reconstruction is small, and the contribution of this assumption is controlled via the magnitude of the regularization parameter. Even though TV regularization is known to be robust in noise removal while preserving edge information in sparse data reconstructions, it causes the resulting image to appear blurred because of the way it performs its computation. This blurring affects the performance of the segmentation algorithm, especially for greatly reduced data with lots of noise to regularize. This is likely the reason why parameters obtained with TV regularization were not as close to the reference data as those obtained with DART. As such, the *a priori* knowledge used by TV regularization may not be ideal for µCT imaging of bone, where a lot of small details need to be preserved. Due to the ill-posed nature of tomographic reconstruction, some *a priori* knowledge is needed in very sparse cases, as can be seen from the poor performance of CGLS for extreme cases. The discrete *a priori* knowledge incorporated by DART seems to be strongly preferable in this application, in which the data itself is nearly discretely distributed to several classes.

DART was the only algorithm capable of preserving the statistically significant differences in BV/TV, Tb.Th and EF across all data reduction levels, and it did not give rise to extra parameters becoming significantly different. However, the Tb.Th values with one-fourth and one-sixth of the original data became significant also with the two-tailed test, while they were only significant with the one-tailed test in the original data. With CGLS and TV, significant differences were only preserved for BV/TV and EF when the original dataset was reduced to half. Reduction to one-quarter of the data preserved the statistically significant differences in BV/TV and EF with CGLS, and only in EF with TV. Additionally, TV generated unwanted statistically significant results for Tb.N. For a reduction to one-sixth of the original projection data, CGLS and TV both preserved the significant difference in EF, but generated incorrect significant differences in Tb.N. These results support the notion that DART was the most reliable of the three IR algorithms analyzed in this study.

The IR algorithms take more computing time than the FDK algorithm. For CGLS, the computational times were almost two times the computing times of FDK when handling half of the projection data, but remained in an acceptable range because they were still in the order of tens of seconds with our workstation. However, when using TV and DART, the computations took considerably longer. Time inefficiency may be tolerated, though, since TV and DART require much less input data for successful reconstruction of bone geometry, resulting in reductions of imaging time and radiation dose. Furthermore, less data storage is required, which may be of value for some situations. For these reasons, IR methods facilitate the imaging of large sample series, as more scientifically relevant data can be acquired per unit of time and computer memory.

Regarding CNR, TV expressed the highest values across all reduced datasets. The texture in TV reconstructed images is typically smooth and contains little noise, so it is expected to have better values in basic metrics of image quality compared to CGLS and DART. CGLS and DART performed similarly to FDK, with DART having slightly lower CNR values. The lower CNR values are likely caused by the appearance of small speckles in the background sometimes seen in DART reconstructions. This artifact did not occur in the segmentation results. It should be noted that achieving high absolute image quality was not the primary aim of this research, therefore traditional image quality metrics may not be the best way in drawing conclusions from our data. Instead, we wanted to obtain reconstructions of sufficient quality so that reliable segmentations of bone and other tissues are possible, and quantitative bone analyses can be performed reliably from reduced datasets.

While the results suggest that collection of less projection data in µCT is feasible when the reconstruction is done iteratively, the study had the following limitations. While we chose data reconstructed with FDK as the reference method in our study, it should be noted that it does not output exact ‘ground truth’ data due to approximation errors caused by the cone-beam imaging geometry. It is, however, the most commonly used reconstruction algorithm in µCT studies as commercial scanners routinely output FDK data. Since we studied limited-data solutions to µCT imaging, using FDK as the reference is a rational and practical choice. Furthermore, the reconstruction framework we used assumed a point source in computing the system matrix for reconstruction. The scanner we used, however, has a nonzero focal spot size, thus the assumption may have produced minor error in the computations. Finally, although we used an analytical method and three distinct iterative algorithms in this study, there remain many different algorithms that could have been used. In the future, additional methods could be tested that are not discussed here, including but not limited to entropy methods^53^, multiplicative algebraic methods^54^, statistical inversion^55^, and deep-learning based approaches^56^.

In conclusion, we demonstrated that iterative and regularizing image reconstruction algorithms applied to reduced projection data are sufficiently reliable when used for morphological bone analysis involving segmentation. Furthermore, we were able to quantify changes in bone structure in early OA using sparse projection data. Thus, there is the potential for iterative reconstruction algorithms to replace the algorithms presently considered the gold-standard in micro-structural analyses of bone. In particular, discrete methods, such as DART, seem promising in quantitative parameter analyses requiring segmented data. By reconstructing data with iterative methods, the amount of projection data needed can be drastically lowered, thereby reducing imaging times and radiation doses associated with µCT imaging. This would allow for a larger number for imaged samples in *in vitro* studies, and prevent harmful radiation effects in *in vivo* studies.
